# TCRβ Sequencing Reveals Spatial and Temporal Evolution of Clonal CD4 T Cell Responses in a Breach of Tolerance Model of Inflammatory Arthritis

**DOI:** 10.3389/fimmu.2021.669856

**Published:** 2021-04-27

**Authors:** Shaima Al Khabouri, Robert A. Benson, Catriona T. Prendergast, Joshua I. Gray, Thomas D. Otto, James M. Brewer, Paul Garside

**Affiliations:** Institute of Infection, Immunity and Inflammation, College of Medical, Veterinary and Life Sciences, University of Glasgow, Glasgow, United Kingdom

**Keywords:** TCRβ sequencing, T cell clonality, CD4 T cell, rheumatoid arthritis, tolerogenic therapy

## Abstract

Effective tolerogenic intervention in Rheumatoid Arthritis (RA) will rely upon understanding the evolution of articular antigen specific CD4 T cell responses. TCR clonality of endogenous CD4 T cell infiltrates in early inflammatory arthritis was assessed to monitor evolution of the TCR repertoire in the inflamed joint and associated lymph node (LN). Mouse models of antigen-induced breach of self-tolerance and chronic polyarthritis were used to recapitulate early and late phases of RA. The infiltrating endogenous, antigen experienced CD4 T cells in inflamed joints and LNs were analysed using flow cytometry and TCRβ sequencing. TCR repertoires from inflamed late phase LNs displayed increased clonality and diversity compared to early phase LNs, while inflamed joints remained similar with time. Repertoires from late phase LNs accumulated clones with a diverse range of TRBV genes, while inflamed joints at both phases contained clones expressing similar TRBV genes. Repertoires from LNs and joints at the late phase displayed reduced CDR3β sequence overlap compared to the early disease phase, however the most abundant clones in LNs accumulate in the joint at the later phase. The results indicate CD4 T cell repertoire clonality and diversity broadens with progression of inflammatory arthritis and is first reflected in LNs before mirroring in the joint. These observations imply that antigen specific tolerogenic therapies could be more effective if targeted at earlier phases of disease when CD4 T cell clonality is least diverse.

## Introduction

Rheumatoid arthritis (RA) is a chronic inflammatory autoimmune disease characterised by synovial inflammation and cartilage and bone erosion, causing progressive loss of joint function (1). Antigen presentation and CD4 T activation mechanisms have been shown to play a role in the pathogenesis of RA. This is evidenced by genetic association studies in RA patients showing strong associations of HLA-DRB alleles, T cell activation associated genes such as *PTPN22* and *CTLA-4*, and loci associated with signal transduction such as *STAT4* (2–7). Moreover, the successes of therapeutics in modulating T cell activation, such as the use of Abatacept, reinforce the importance of CD4 T cells in propagating the disease (8). As such, there has been increasing interest in targeting pathogenic autoreactive CD4 T cells using antigen specific tolerogenic therapies as this line of therapy aims to re-establish tolerance and provide long-lasting remission, while retaining protective immunity against pathogens. However, targeting autoreactive CD4 T cells has proven difficult in RA, due to the broad range of antigens implicated in disease, lack of a clear cellular hierarchy of disease drivers, and also patient to patient variation (9–13). Moreover, it is unknown at what stages of the disease these autoreactive responses develop nor the location in which these responses are primed, thus hampering the development of effective tolerogenic therapies. Antigen specific responses can be identified by detecting expanded clonal T cell populations. Indeed, oligoclonal CD4 T cell responses and skewed TCR repertoires have been reported in arthritic joints (14–17). More recently, analysis of CD4 T cell behaviour was shown to reflect antigen recognition in inflamed joints at the early stages of disease (18), suggesting that antigen specific CD4 T cell responses may perpetuate and drive progression of RA. However, the development of antigen specific responses and their evolution with the progression of RA is poorly understood. Investigating the evolution TCR clonality of antigen experienced CD4 T cells in RA will provide insight on how antigen specific responses evolve with disease progression. This will inform the development of more effective tolerogenic therapies by indicating the range of clones that would need to be targeted and the disease stage at which these therapies are most likely to be effective. In this study, we employed breach of self-tolerance models of antigen induced inflammatory arthritis (19, 20) and chronic polyarthritis (21) in which autoreactive responses develop. These models also recapitulate the early and later stages of the disease – hereafter named the early and late phases respectively. TCRβ sequencing was used to monitor the evolution of antigen specific endogenous CD4 T cell responses in inflamed joints and their associated draining lymph nodes.

## Methods

### Animals

Male and female C57BL/6 mice aged 7-12 weeks were purchased from Envigo (UK), and used as adoptive transfer recipients. OT-II TCR transgenic mice (22) were bred inhouse. All mice were maintained at the University of Glasgow’s central research facility and housed under standard housing conditions specified by the UK Home Office.

### Induction of Inflammatory Arthritis

“Early” antigen induced inflammatory arthritis was conducted as previously described (19, 20). Briefly, 2-3x10^6^ Th1 polarised OT-II transgenic T cells were transferred i.v. into C57BL/6 recipients. Recipients were immunised with 100µL of 1µg/mL of OVA protein (Worthington Biochemicals) in Freund’s complete adjuvant (CFA) (Sigma-Aldrich) subcutaneously after 24 hours. Ten days later, mice were given a periarticular injection in footpads with 50µL of 100µg of heat aggregated OVA (HAO) or PBS as a control. Mice were sacrificed four days later and cells were isolated from joint tissue and draining popliteal lymph nodes (pLN).

The “late” model antigen induced inflammatory arthritis was modified from a model of chronic polyarthritis (21). 30 days after HAO administration described above, mice were given a second periarticular injection of 100µg of HAO in incomplete Freund’s adjuvant (IFA) (Sigma-Aldrich) or IFA alone as a control. Mice were sacrificed 17 days later and cells were isolated from joint tissues and pLNs.

### Isolation of Cells From Tissues

Cells from mouse joints and pLNs were isolated as previously described (18). Briefly, ankle joints were teased apart and shaken at 110 rpm at 37°C for 25 minutes with 2.68mg/mL collagenase D (Roche) in RPMI 1640 (Gibco, Thermo Fisher Scientific). Joint tissue was then homogenised using a gentleMACS Dissociator (Miltenyi Biotech) then strained to obtain single cell suspensions. PLNs were forced through Nitex mesh (Cadisch Precision Meshes) to obtain single cell suspensions. Samples were washed and stained for flow cytometry. Joint and pLN samples were analysed separately. Joint samples and pLN samples were pooled per mouse.

### Flow Cytometry

Cells were stained for flow cytometry as described previously (18). The following antibodies were used: anti-CD4 (clone GK1.5), anti-CD44 (clone IM7), anti-CD45 (clone 30-F11), anti-CD45.1 (clone A20). Data was analysed using FlowJo v10 software (Treestar, Oregon USA).

### TCRβ Sequencing

The data for this study have been deposited in the European Nucleotide Archive (ENA) at EMBL-EBI under the accession number PRJEB40509 (https://www.ebi.ac.uk/ena/browser/view/PRJEB40509). CDR3β sequencing was performed by iRepertoire Inc. (Huntsville, AL, USA) on total RNA collected from sorted antigen experienced endogenous CD4 T cells (CD4+, C45.1-, CD44hi). CD4+, CD45.1-, CD44hi were sorted from joint and pLN samples using the BD Aria III FACS sorter (BD Biosciences). Samples were collected separately from individual mice in lysis buffer (Purelink RNA micro kit, Thermofisher) + 1% β-mercaptoethanol (Sigma-Aldrich) and lysed using 29G insulin syringes (VWR). Lysed cells were then stored at -80°C prior to RNA purification. Total RNA was purified using the Purelink RNA micro kit (Thermo Fisher Scientific) as per manufacturer’s instructions and quality assessed by measuring A260/280 using a Nanodrop 1000 spectrophotometer (Thermo Fisher Scientific). Barcoded libraries were prepared per sample using barcoded primers covering the V-J TCR region, then amplified, pooled, and sequenced using the Illumina MiSeq platform covering 150 paired end reads (PER). Basic data analysis was performed by iRepertoire. Sequencing data was also prepared for analysis using MiXCR developed by Bolotin et al. (23) and analysed using tcR developed by Nazarov et al. (24) in the R statistical software frame work (R version 3.4.3).

### Calculation of the D50 Diversity Index (DI)

Diversity was calculated using iRepertoire’s data analysis guide. For samples where the number of unique CDR3β sequences ≥10,000:

DI = rank of unique CDR3β sequence where 50% of the top 10,000 total reads falls × 100/10,000

For samples where the number of unique CDR3β sequences <10,000:

DI = rank of unique CDR3β sequence where 50% of the total reads falls × 100/no. of unique CDR3β sequences

### Calculation of Repertoire Overlap

The number of shared CDR3β amino acid sequences is represented as a normalised overlap index taking into consideration the size of the repertoires being compared. The normalised overlap index was calculated using the tcR R package (24) using the following calculation:

Normalised overlap index = No. of exact overlapping CDR3β amino acid sequences/total reads repertoire 1 × total number of reads repertoire 2

### Statistics

Data is shown as mean ± SD. Specific test and significance levels are stated in respective figure legends. Statistical analyses were performed using GraphPad Prism version 7 (GraphPad Inc, CA, USA) or using the R statistical software framework (R version 3.4.3).

## Results

### Induction of Inflammatory Arthritis Results in Local Accumulation of Endogenous Antigen Experienced CD4 T Cells at Both the Early and Late Phases

**Figure 1:**
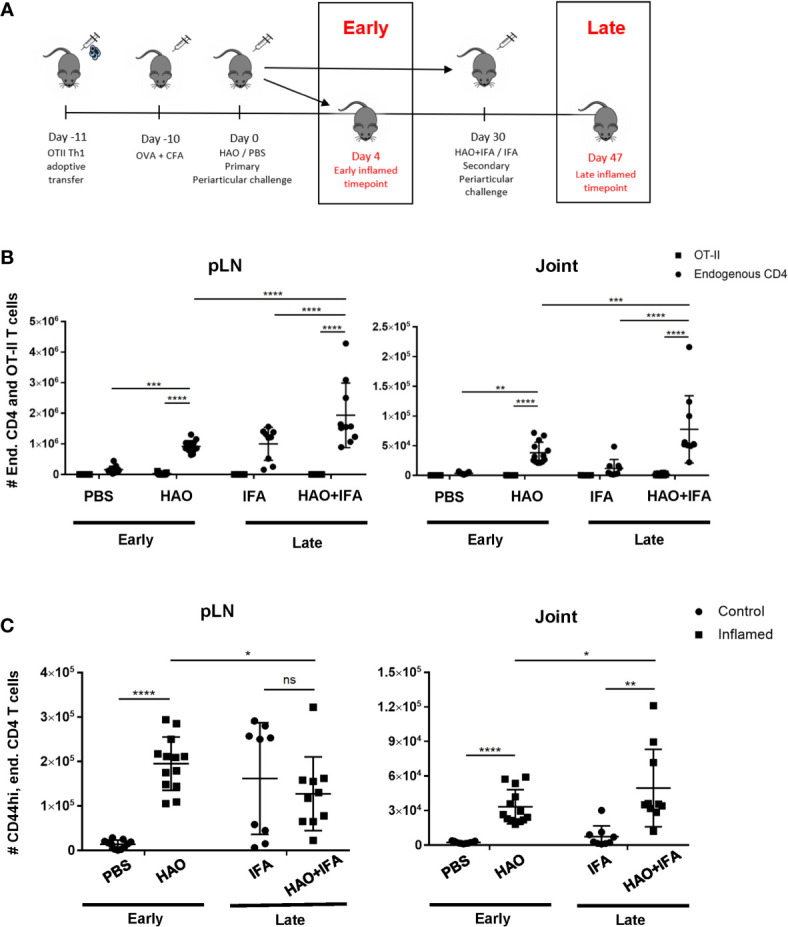
Outline of model phases and number of endogenous, OT-II, and endogenous antigen experienced CD4 T cells at both phases. **(A)** Illustration of early and late phases of inflammatory arthritis. **(B)** Number of endogenous CD4 T cells (CD4+, CD45+, CD45.1-) and OT-II T cells (CD4+, CD45+, CD45.1+) from pLNs and joints of mice undergoing inflammatory arthritis at the early and late phases and respective controls. **(C)** Number of antigen experienced endogenous CD4 T cells (CD4+, CD45+, CD45.1-, CD44hi) from pLNs and joints of mice undergoing inflammatory arthritis at the early and late phases and respective controls. Data is representative of mean ± SD with each point representing individual experimental mice. Data represents three independent experiments for the early phase and two independent experiments for the late phase with n=5 for each experiment. Groups were compared using 2-way ANOVA and Student’s t-tests. Stars represent the following p-values: *<0.05, **<0.01; ***<0.001; ****<0.0001; ns, not significant. HAO, heat-aggregated OVA; IFA, incomplete Freund’s adjuvant.

Induction of the breach of tolerance model of inflammatory arthritis requires the transfer of Th1 polarised ovalbumin (OVA) specific OT-II cells and subsequent immunisations with OVA and heat aggregated OVA (HAO) as outlined in **Figure 1A**. This results in the influx of the transferred OT-II cells and endogenous CD4 T cells of varying antigen specificities, indicated by the range of expressed TCRβ variable genes (TRBV) (18). Assessing the evolution of TCR clonality of the endogenous CD4 T cell population requires the accurate identification of these cells in joints and popliteal lymph nodes (pLN), being distinguishable from the transferred OT-IIs by expression of the congenic marker CD45.1 **(** **Supplementary Figures 1** and **2** **)**. Endogenous CD4 T cells were present at a significantly higher number than the transferred OT-II cells in inflamed pLNs and joints at both the early and late phases **(** **Figure 1B** **)**. A significant number of endogenous CD4 T cells at the early phase also displayed an antigen experienced phenotype, indicated by increased CD44 expression, after induction of inflammatory arthritis with HAO **(** **Figure 1C** **)**, and were previously shown to produce TNFα and IFNγ following ex-vivo stimulation with PMA/ionomycin (18). The number of endogenous CD4, CD44hi cells was also increased at the late phase in inflamed joints **(** **Figure 1C** **)**.

### Accumulation of Expanded Clones and Observed Clonality and Diversity of CD4 T Cell Repertoires Is Antigen Driven

**Figure 2:**
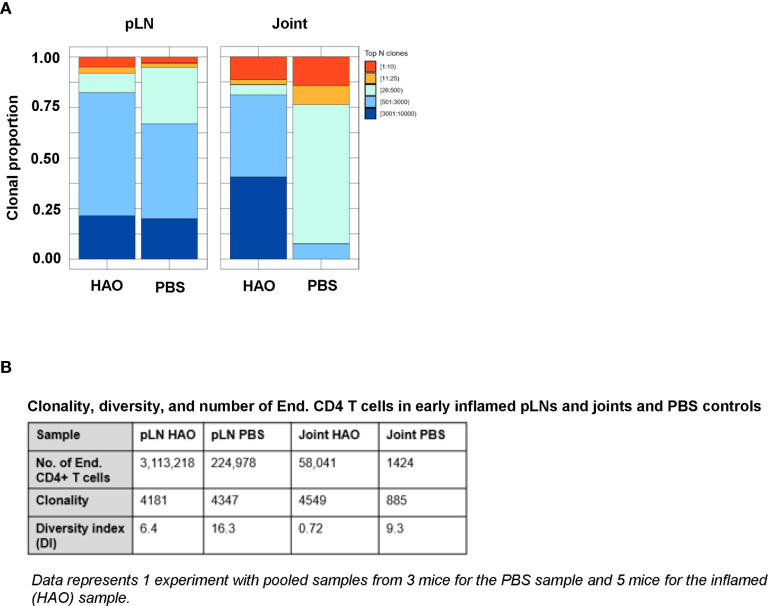
Characterisation of antigen experienced endogenous CD4 T cell repertoires from inflamed pLNs and joints and PBS controls at the early phase. Proportion of the top 10 most frequently occurring clones, followed by the next 25, 500, 3000, 10,000, and 100,000 most frequent clones contributing to the endogenous antigen experienced CD4+ T cell repertoire in **(A)** pooled inflamed pLNs and joint samples at the early phase (HAO) and pooled PBS controls. **(B)** Descriptive statistics of pooled inflamed pLN and joint samples at the early phase and their PBS controls indicating endogenous CD4 T cell number, clonality, and diversity. Data represents 1 experiment with pooled samples from 3 mice for the PBS sample and 5 mice for the inflamed (HAO) sample.

To investigate the evolution of CD4 TCR clonality in this model of inflammatory arthritis, endogenous antigen experienced CD4 T cells i.e. excluding the transferred OT-II cells, were sorted from pLNs and joints of individual mice at both early and late phases. Total RNA was then isolated from the endogenous CD4 T cells and used to sequence the CDR3β region of the TCR, the most variable region of the TCR critical in recognition of specific peptide sequences (25, 26). Repertoires were then characterised by examining the number of unique CDR3β sequences and evaluating the degree expanded clones contributed to the overall antigen experienced repertoire. From this, the clonality and diversity of the CD4 T cell repertoire can be determined. In this study, clonality is defined by the number of unique CDR3β sequences found in the population, and diversity—represented by the Diversity Index (DI) (see *Methods*)—represents the relative contribution of each unique CDR3β sequence, or clone, to the population. Thus, a repertoire with a larger number unique CDR3β sequences, and therefore clones, would be described to have high clonality. This repertoire would also display high diversity and have a high DI value if the number of cells contributing to different clonal populations are more evenly distributed. Conversely, the repertoire would be described to have low diversity and have a low DI value if the repertoire is mainly comprised of a few clones present in high frequencies relative to the total number of clones. Due to the low recovery of antigen experienced endogenous CD4 T cells isolated from control pLNs and joints at the early phase, cells from control pLNs or joints needed to be pooled prior to sequencing. Pooling samples changes the composition of the repertoire and affects T cell repertoire diversity, so comparisons of control CD4 T cell populations were made against pooled data from inflamed joint and pLN samples at the early phase **(** **Figures 2A, B** **)**.

Clonality of the endogenous CD4 T cell repertoire from inflamed pLNs at the early phase and controls were comparable (4181 unique CDR3β sequences in the inflamed pLN repertoire vs 4347 in the PBS pLN repertoire). However, the repertoire from the inflamed pLN showed a reduced diversity compared with the control pLN sample (6.4 vs 16.3 respectively). Endogenous CD4 T cell repertoires from inflamed joints displayed higher clonality than controls (4549 vs 885). Despite this, the diversity of the repertoire in the inflamed joint was relatively low compared to the PBS joint (0.72 vs 9.3 respectively). These data therefore demonstrate that accumulation of clones can be detected at the early phases of the disease in both inflamed pLNs and joints.

**Figure 3:**
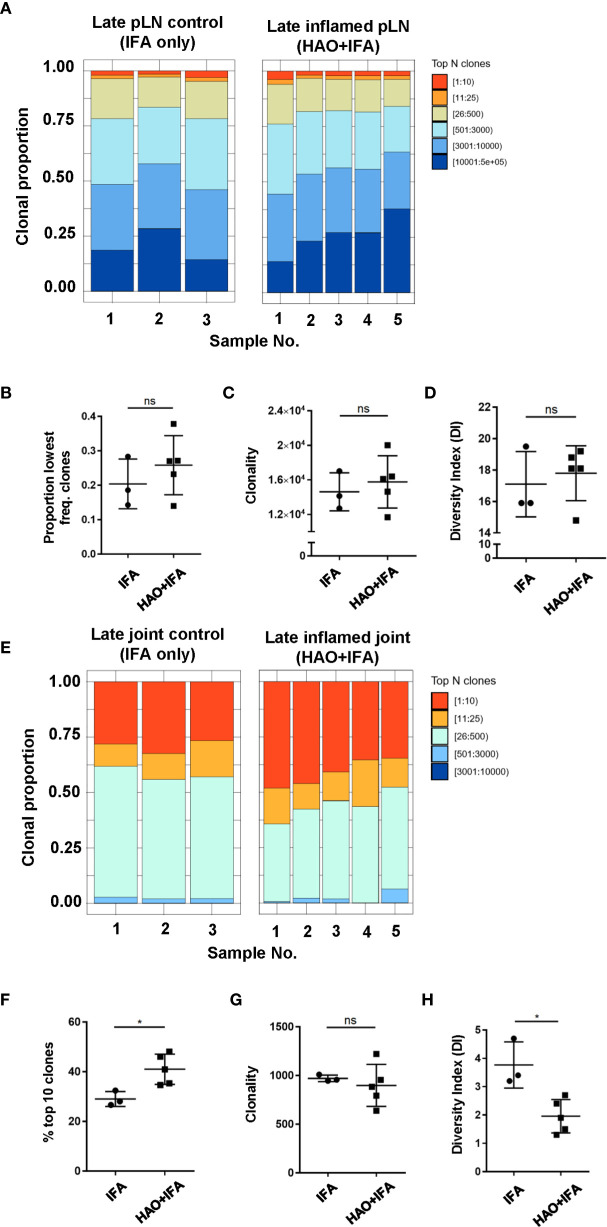
Characterisation of antigen experienced endogenous CD4 T cell repertoires from inflamed pLNs and joints and IFA controls at the late phase. Proportion of the top 10 most frequently occurring clones, followed by the next 25, 500, 3000, 10,000, and 100,000 most frequent clones contributing to the endogenous antigen experienced CD4+ T cell repertoire in **(A)** inflamed pLNs, and **(E)** inflamed joints at the late phase (HAO+IFA) and IFA only controls. **(B)** Proportion of the least frequently occurring clones in inflamed pLNs at the late phase and IFA controls. **(F)** Percentage contribution of the top 10 most frequently occurring clones in inflamed joints at the late phase and IFA controls. **(C**, **G)** clonality, represented as the number of unique CDR3β DNA sequences, in **(C)** inflamed pLNs and IFA controls, and **(G)** inflamed joints and IFA controls at the late phase. **(D**, **H)** diversity indices of **(D)** inflamed pLNs and IFA controls, and **(H)** inflamed joints and IFA controls at the late phase. Data is representative of mean ± SD with each point representing individual experimental mice. Data represents 1 experiment with n=5 for the inflamed group and n=3 for the control group. Groups were compared using unpaired Student’s t-test. Stars represent the following p values: *<0.05; ns, not significant.

Evidence for the accumulation of antigen experienced T cells in the absence of their cognate antigen has been demonstrated in this model (18) and in RA patients (27–29) which may be a result of a chronic inflammatory environment (30, 31). To address whether the accumulation of clones is antigen driven rather than a result of non-antigen induced inflammation, the antigen experienced repertoires of pLNs and joints at the late phase were compared to repertoires isolated from mice challenged with IFA alone **(** **Figure 3A** **)**, where inflammation is induced without the presence of antigen. No significant difference was found between the contribution of the top 10 most expanded clones to the repertoires of inflamed pLNs at the late phase and IFA controls (data not shown) nor when comparing the contribution of clones occurring at the lowest frequency **(** **Figure 3B** **)**. The number of clones occurring at a frequency of 2 or more were also compared between inflamed and IFA pLN samples as this was the median frequency of clones found in the PBS pLN sample (data not shown). Thus, clonal populations found at higher than this frequency are assumed to have accumulated due to the presence of antigen. No clonal accumulation was detected between inflamed pLNs at the late phase and IFA controls (*p value 0.32 Welch two sample t-test*). Moreover, repertoires from inflamed pLNs at the late phase and IFA displayed comparable levels of clonality and diversity **(** **Figures 3C, D** **)**.

In late phase arthritic joints, the top 10 highly expanded clones constituted approximately 41% of the overall repertoire compared to joint IFA controls, where the top clones only comprised an average of 29% of the entire repertoire **(** **Figures 3E, F** **)**. No difference in clonality was detected between repertoires from inflamed joints and IFA controls **(** **Figure 3G** **)**, but inflamed joints have reduced clonal diversity compared to IFA controls **(** **Figure 3H** **)**. This would suggest that antigen driven accumulation of expanded clones in inflamed joints can also be observed at the late phase.

### The Antigen Experienced Endogenous CD4 T Cell Repertoire Displays Disparity in Clonal Diversity Between Inflamed pLNs and Joints With Time

**Figure 4:**
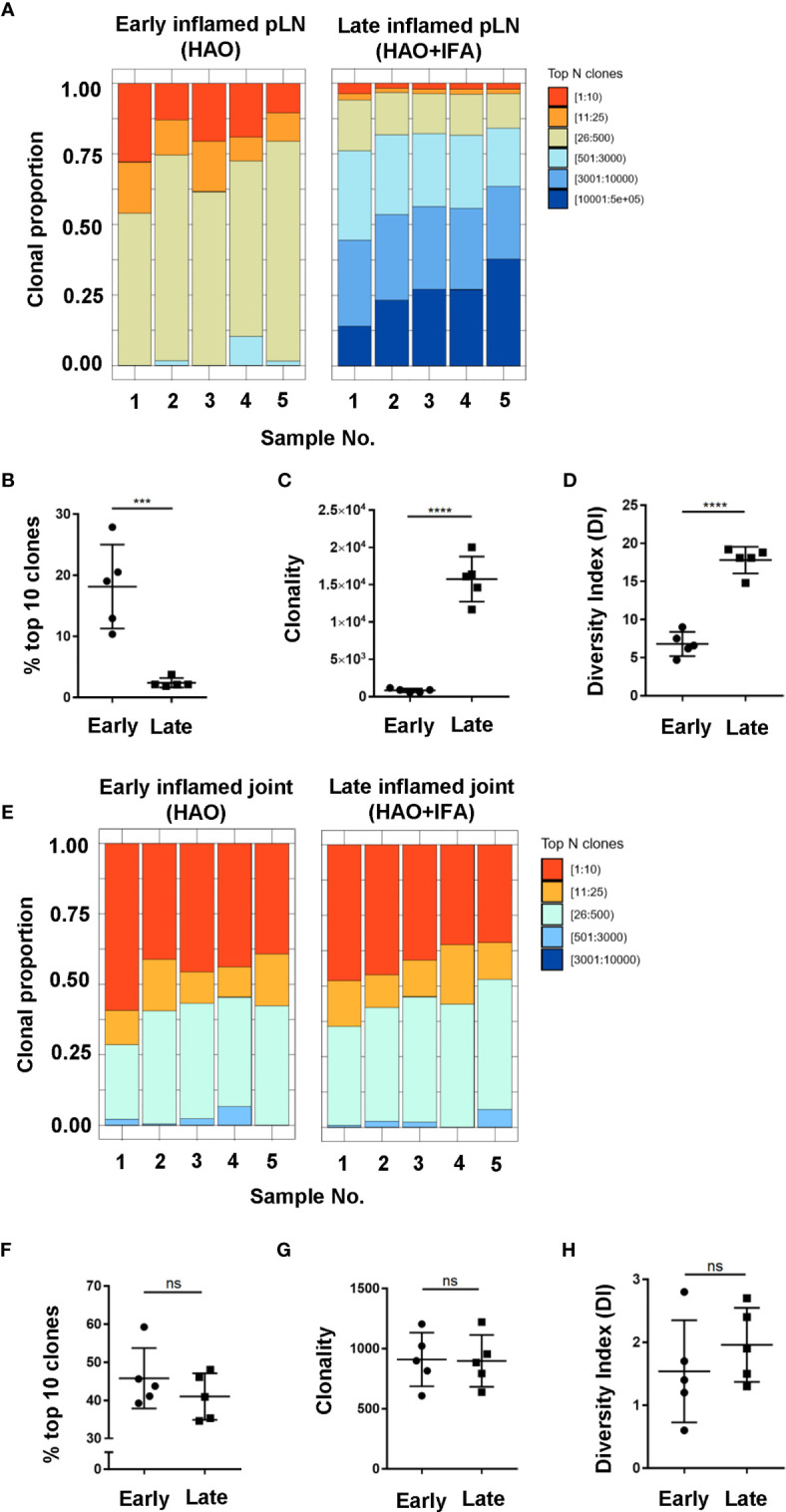
Characterisation of antigen experienced endogenous CD4 T cell repertoires in inflamed pLNs and joints at the early and late phases. Proportion of the top 10 most frequently occurring clones, followed by the next 25, 500, 3000, 10,000, and 100,000 most frequent clones contributing to the endogenous antigen experienced CD4+ T cell repertoire in **(A)** inflamed pLNs at the early and late phases, and **(E)** inflamed joints at the early and late phases. **(B**, **F)** percentage contribution of the top 10 most frequently occurring clones in **(B)** inflamed pLNs and **(F)** inflamed joints at the early and late phases. **(C**, **G)** clonality, represented as the number of unique CDR3β DNA sequences, in **(C)** inflamed pLNs, and **(G)** inflamed joints at the early and late phases. **(D**, **H)** diversity indices of **(D)** inflamed pLNs, and **(H)** inflamed joints at the early and late phases. Data is representative of mean ± SD with each point representing individual experimental mice. Data represents 1 experiment with n=5 for both the early and late phase groups. Groups were compared using unpaired Student’s t-test. Stars represent the following p values: ***<0.001; ****<0.0001; ns, not significant.

Antigen experienced endogenous CD4 T cell repertoires from pLNs and joints were compared between the early and late phases to investigate how CD4 TCR clonality evolves with disease progression **(** **Figures 4A, E** **)**. When comparing inflamed pLNs at the early and late phases, the top 10 most expanded clones contributed approximately 18% to the overall antigen experienced repertoire of inflamed pLNs at the early phase compared to only 2% at the late phase **(** **Figures 4A, B** **)**. When comparing clonality and diversity, repertoires from pLNs isolated at the late phase displayed increased clonality and diversity compared with their early counterparts **(** **Figures 4C, D** **)** and comprised mainly of low abundance clones. In contrast to the pLNs, antigen experienced CD4 T cell repertoires isolated from inflamed joints at the early and late phases were similar; the top 10 most expanded clones dominated the repertoires and comprised on average 46% and 41% of the overall populations **(** **Figures 4E, F** **)**. Furthermore, no differences were observed between the clonality and diversity of the repertoires at the early and late phases **(** **Figures 4G, H** **)** and repertoire diversity overall was low in the joint at both phases. Together, these data highlight the disparity in repertoire composition between pLNs and joints at the early and late phases, namely that pLNs at the late phase displayed high repertoire clonality and diversity compared to their early counterparts and also compared to inflamed joints at the same phase.

### Disparity in Clonality of the Antigen Experienced Endogenous CD4 T Cell Repertoires Is Attributed to Accumulation of Clones With Different TCRβ Variable (TRBV) Genes

**Figure 5:**
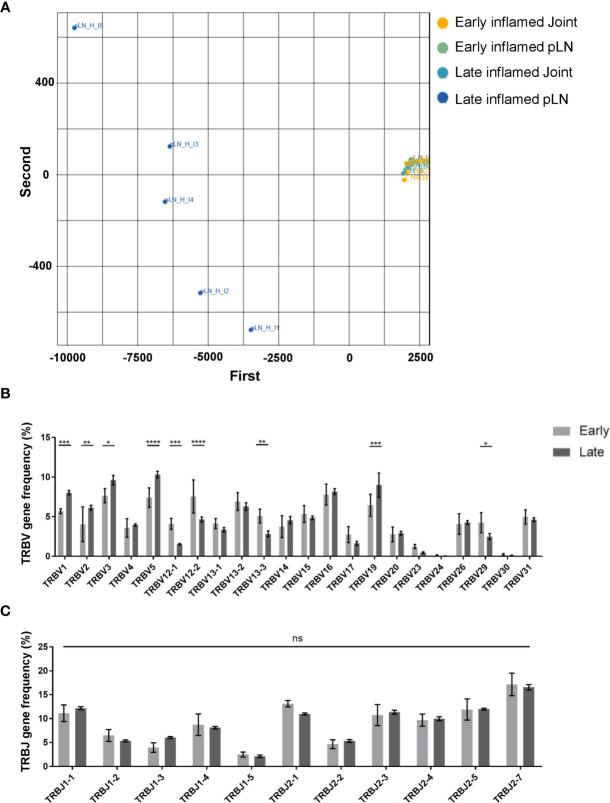
Range of TRBV genes expressed in antigen experienced endogenous CD4 T cell repertoires of inflamed pLNs and joints at the early and late phases. **(A)** PCA plot of TRBV gene frequency in repertoires of inflamed pLNs and joints at the early and late phases. Frequency of **(B)** TRBV gene expression, and **(C)** TRBJ gene expression in inflamed pLNs at the early and late phases. Graphs represent 1 experiment with n=5 for both the early and late inflamed groups. Groups were compared using ordinary 2-way ANOVA with Sidak’s multiple comparisons test. Data presented as mean ± SD. Stars represent the following p values: *<0.05; **<0.01; ***<0.001; ****<0.0001; ns, not significant.

Characterisation of the antigen experienced CD4 T cell repertoires from pLNs and joints at the early and late phases provided useful information on the overall distribution of clones in these repertoires. However, this does not provide any insight on the TCR sequence of these clones, nor on whether these clones have similar or different TCR sequences between the two sites. Moreover, no insight is gained on whether the clonal composition of the antigen experienced repertoires in these two sites change over time. To address this, a PCA analysis was performed on inflamed pLNs and joints at both the early and late phases on the basis of TRBV gene use **(** **Figure 5A** **)**. Repertoires isolated from inflamed pLNs at the late phase displayed the largest range in TRBV gene use, while inflamed pLNs and joints at the early phase as well as inflamed joints at the late phase grouped together in terms of TRBV gene expression. Indeed, when quantifying these differences, inflamed pLNs at the late phase had significantly increased presence of TRBV genes 1, 2, 3, 5, and 19, while TRBV genes 12-1, 12-2, and 13-1 were present in the highest frequencies in inflamed pLNs at the early phase **(** **Figure 5B** **)**. These differences are also due to changes in TRBV gene use and not in TRBJ genes **(** **Figure 5C** **)**. Interestingly, the PCA analysis also highlighted inter-sample differences in TRBV gene use in inflamed pLNs at the late phase. Accumulation of clones expressing different TRBV genes in inflamed pLNs at the late phase suggests changes in antigen specific responses with time and also between inflamed pLNs and their associated tissues. Moreover, these changes in antigen specific responses also differ between individual animals with time.

### Antigen Associated Changes in the Inflamed pLN May Predict the Changes in Antigen Specificities and Clonality of the CD4 T Cell Repertoire in Inflamed Joints

**Figure 6:**
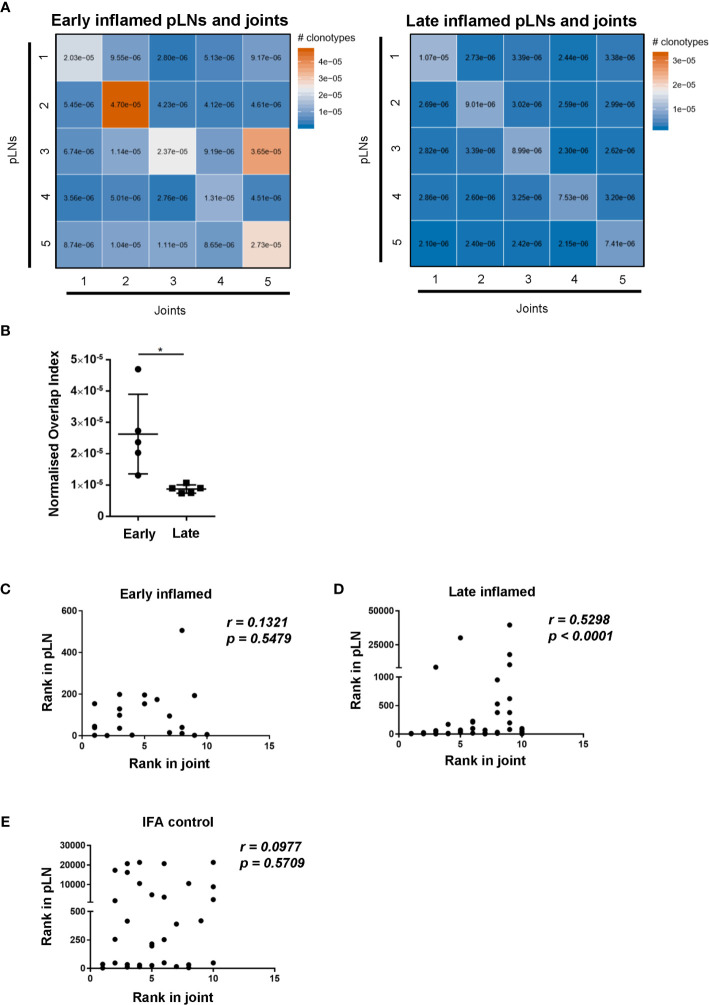
Degree of CDR3β amino acid sequence overlap and correlation of most abundant clones between inflamed pLNs and joints at the early and late phases. **(A)** Heatmaps of normalised number of CDR3β amino acid sequences (see methods) between inflamed pLNs and joints at the early (left) and late (right) phases. **(B)** Graph representing the intersects (diagonal) of **(A)** representing CDR3β sequence overlap between inflamed pLNs and joint within the same animal. **(C–E)** Correlation of the top 10 ranked clones in joints with their rank in respective pLN samples. The rank of each of the top 10 clones in **(C)** inflamed joint at the early phase, **(D)** inflamed joint at the late phase, and **(E)** IFA control joint samples were plotted against the rank of that clone in respective pLN samples. Data is representative of one experiment with n=5 for the early and late phase inflamed groups and n=3 for controls. Graph in **(B)** is representative of mean ± SD with each point representing individual experimental mice. * represents p<0.05. In C-E correlations (r) were calculated using Spearman’s correlation after testing for normality of the data using the D’Agostino-Pearson test. p represents the p value.

To assess whether changes in CD4 TCR clonality reflected changes antigen specificities, the degree of CDR3β amino acid sequence overlap between the antigen specific CD4 T cell repertoires was investigated. Comparing the repertoire overlap between inflamed pLNs and joints at both the early and late phases allowed us to monitor clonal dynamics between these two sites with the progression of inflammatory arthritis. The number of overlapping CDR3β amino acid sequences is represented by the normalised overlap index (see methods) and this decreased between the inflamed pLN and its joint with the progression of inflammatory arthritis **(** **Figures 6A, B** **)**. CDR3β sequence overlap decreased significantly between samples when comparing repertoires from inflamed pLN samples at the early phase to inflamed pLNs at the late phase **(** **Supplementary Figure 3A** **)**, while the degree of CDR3β amino acid sequence overlap remained unchanged between repertoires from inflamed joints at the early and late phases **(** **Supplementary Figure 3B** **)**. These observations confirm the observed changes in TRBV gene use and also imply changes in antigen specific responses in pLNs, which progress towards polyclonality with disease progression. Correlation analysis revealed that the top 10 most expanded clones in the inflamed joint at the late phase correlated significantly with the most expanded clones in the inflamed pLNs at the same phase **(** **Figure 6D** **)**. Furthermore, the 10 most expanded clones in inflamed joints at the early phase and those from IFA controls did not correlate with the most highly expanded clones in their respective pLNs **(** **Figures 6C, E** **)**. Moreover, the 10 most expanded clones in inflamed joints at the early phase were not all present in their respective pLNs **(** **Supplementary Table 1** **)**, but the top 10 expanded clones in inflamed joints at the late phase were all present in inflamed pLNs at the same phase **(** **Supplementary Table 2** **)**. These results indicate that despite pLNs progressing towards polyclonality with the development of disease, the most highly expanded antigen experienced clones accumulate in the inflamed joint. Thus repertoire clonality in the pLN may eventually be mirrored in the inflamed joint with disease progression.

## Discussion

Characterising CD4 TCR clonality has been used to monitor progression of disease states in cases of infection (32) and autoimmunity (33, 34), including RA (15, 35–38). Although CD4 T cell clonality has been determined in RA patients at different stages of the disease (15, 17), the development of antigen specific responses from very early, pre-clinical stages to a more established disease stage remains unknown. Understanding this is important to develop effective tolerogenic therapies as the range of CD4 T cell clones to target and the timepoint this therapy will have the greatest impact can be determined. Thus, we investigated the evolution of CD4 TCR clonality by sequencing CDR3β regions of antigen experienced endogenous CD4 T cells isolated from pLNs and joints at early and late phases of breach of self-tolerance models of inflammatory arthritis.

Induction of inflammatory arthritis resulted in the accumulation of endogenous CD4 T cells displaying an antigen experienced phenotype. This population has been previously shown to produce TNFα and IFNγ and also harbour autoreactive clones capable of causing bone and cartilage degradation (18–21, 39, 40). CDR3β analysis of this this population revealed that TCR repertoires from inflamed joints at both the early and late phases displayed low clonality and diversity and were dominated by few clones present at high frequencies. In contrast, pLNs at the late phase displayed higher clonality and diversity compared with their counterparts at the early phase and were mainly composed of a large number of clones that were present at low frequencies. This indicates that pLNs become more polyclonal with the progression of inflammatory arthritis. Moreover, correlation of the most frequently occurring clones in the inflamed joint at the late phase with the most frequently occurring clones in the inflamed pLN at the same phase indicates that this polyclonality occurs in the pLNs before possibly being reflected in inflamed joints. Addressing whether the joint endogenous CD4 T cell repertoire does in fact become more polyclonal with more established disease was not possible due to the self-resolving nature of the model. However, the observations reported here are in line with clinical studies that have reported increased T cell clonality and diversity in inflamed joints of patients with established RA compared to early RA patients (15, 41).

Uncovering the underlying factors contributing to CD4 T cell repertoire polyclonality was beyond the scope of the study. However, PCA analysis of TRBV genes of repertoires isolated from inflamed pLNs and joints at the early and late phases highlighted the expanded range of TRBV genes present in repertoires of pLNs at the late phase, indicating a possible change in antigen specific responses with the progression of inflammatory arthritis as a reason for polyclonality. One theory for changes in antigen driven responses could be due to the release of neo-antigens resulting from continued joint damage and/or epitope spreading, a phenomenon observed in RA patients (42, 43). In the model itself however, polyclonality can also be explained by the re-establishment of lymphatic recirculation. Given that the observations reported were exclusively on the antigen experienced CD4 T cell population, re-establishment of lymphatic recirculation is an unlikely explanation for the changes in the repertoire clonality and diversity observed. Moreover, the 10 most abundant antigen experienced clones correlate significantly between inflamed pLNs and joints at the late phase and not in IFA controls. Inflamed joints were also shown to have reduced diversity in the antigen experienced repertoire compared to joint IFA controls. By taking these observations together, one can deduce that changes in pLN repertoire clonality, diversity, and TRBV genes is in fact antigen driven.

CDR3β amino acid sequence overlap analysis between repertoires isolated from pLNs and joints at early and late phases of inflammatory arthritis highlighted that fewer clones are shared between pLNs and joints at the late phase compared to the early phase. This indicates that antigen specific responses differ between these sites with time, driven by changes in clonality, diversity, and clones with different TRBV genes in the pLN. Moreover, the reduced clonal overlap at the late phase highlight the fact that inflamed pLNs and joints display different antigen specific responses at the same phase. The disparity may highlight a timepoint in the disease where pathogenic clones have not yet migrated to the joint to perform their effector functions and thus provide an opportunity for therapeutic intervention. As such, analysis of CD4 T cell clonality between arthritic lymph nodes and joints could serve as a biomarker for disease state and be a method to stratify different RA patients to better modulate the disease. Assessment of clonality can be performed in conjunction with existing methods used to evaluate changes in arthritic lymph nodes with disease progression, such as using lymph node biopsies to evaluate changes in cellular composition and function at different disease stages (44, 45). Furthermore, as the trend towards polyclonality is likely associated with antigen specific responses, clonal compositions can serve as a biomarker to assess the therapeutic impact of RA tolerogenic therapies (46, 47). Indeed, effective RA therapeutics have been shown to reduce clonal expansion of CD4 T cells (36). In addition, understanding the clonal landscape with disease development can inform when such therapies could be most effective. For instance, antigen specific therapies may be more effective when the CD4 T cell clonality and diversity is restricted.

In conclusion, these data outline the evolution of clonal dynamics between inflamed pLNs and their joints with the progression of inflammatory arthritis in that CD4 TCR clonality, begin by being similar between the two sites then change in the pLN with the progression of the disease before potentially being mirrored in the joint in time. These observations suggest that assessment of CD4 T cell clonality can serve as a biomarker for disease progression, at least in subtypes with significant lymphocyte infiltrate in the synovium, and assess the efficacy of RA therapeutics. Specifically, evaluation of changes in CD4 T cell clonality can be used to assess effective antigen specific tolerogenic therapies in RA and also inform timing of such therapies for maximum therapeutic impact.

## Data Availability Statement

The datasets presented in this study can be found in online repositories. The names of the repository/repositories and accession number(s) can be found below: https://www.ebi.ac.uk/ena/browser/view/PRJEB40509, PRJEB40509.

## Ethics Statement

The animal study was reviewed and approved by The UK Home Office and Glasgow Animal Welfare and Ethical Review Bodies (AWERB).

## Author Contributions

SA and RB designed the research and performed the experiments. SA analysed data, constructed figures, and wrote the manuscript. CP and JG provided technical assistance and helped with analysis. TO provided technical assistance with the sequencing data, and JB and PG designed the research and contributed to writing the paper. All authors contributed to the article and approved the submitted version.

## Funding 

This work was supported by Versus Arthritis. RAB, JMB and PG were supported by the Arthritis Research UK (ARUK) programme grant number 19788 and the Innovative Medicines Initiative EU-funded project Be The Cure (BTCURE) [grant number 115142-2]. JB and PG were also supported by the Arthritis Research UK Rheumatoid Arthritis Pathogenesis Centre for Excellence (RACE) (grant number 20298) and the Research into Inflammatory Arthritis Centre Versus Arthritis (RACE) (grant number 22072). This work has received support from the EU/EFPIA Innovative Medicines Initiative 2 Joint Undertaking RTCure grant no. 777357. SA was supported by the Ministry of Higher Education Oman (MOHE) throughout the study.

## Conflict of Interest

The authors declare that the research was conducted in the absence of any commercial or financial relationships that could be construed as a potential conflict of interest.

## Supplementary Material

Supplementary Figure 1 Gating strategy to identify antigen experienced endogenous CD4 T cells from pLNs and joints. Representative flow cytometry plots identifying antigen experienced endogenous CD4 T cells (CD4+, CD45+, C45.1-, CD44hi) from **(A)** inflamed pLNs, and **(B)** inflamed joints at the early phase of mice undergoing inflammatory arthritis.

Supplementary Figure 2 Representative flow cytometry plots identifying antigen experienced endogenous CD4 T from **(A)** pLN, and **(B)** joint PBS controls at the early phase, **(C)** pLN, and **(D)** joints IFA controls at the late phase, and **(E)** inflamed pLN, and **(F)** inflamed joints at the late phase.

Supplementary Figure 3 Degree of CDR3β amino acid sequence overlap between inflamed pLN and inflamed joint samples at the early and late phases. Heatmaps of normalised number of CDR3β amino acid sequences between **(A)** inflamed pLNs, and **(C)** inflamed joints at the early and late phases. **(B** and **D)** Normalised overlap values between **(B)** inflamed pLN samples, and **(D)** inflamed joint samples at the early and late phases. Data is representative of mean ± SD with each point representing individual experimental mice. Data represents 1 experiment with n=5 for both the early and late inflamed groups. Groups were compared using unpaired Student’s t-test. Stars represent the following p values: ** <0.01; ns: not significant.

Supplementary Table 1 The top 10 frequently occurring clones inflamed joint samples at the early phase ranked in terms of frequency in respective pLNs. The top 10 frequently occurring clones in the antigen experienced CD4+ T cell repertoire were found in each of the inflamed joint samples at the early phase and searched for in respective pLN samples. The CDR3β amino acid sequence, associated TRBV gene, read count, and rank of the clone in the pLN sample was obtained. The rank of each of the top 10 clones in the joint are also included in the table. Data is representative of 1 experiment with n=5.

Supplementary Table 2 The top 10 frequently occurring clones inflamed joint samples at the late phase ranked in terms of frequency in respective pLNs. The top 10 frequently occurring clones in the antigen experienced CD4+ T cell repertoire were found in each of the inflamed joint samples at the late phase and searched for in respective pLN samples. The CDR3β amino acid sequence, associated TRBV gene, read count, and rank of the clone in the pLN sample was obtained. The rank of each of the top 10 clones in the joint are also included in the table. Data is representative of 1 experiment with n=5.
