# Supplementary material for: TCRβ Sequencing Reveals Spatial and Temporal Evolution of Clonal CD4 T Cell Responses in a Breach of Tolerance Model of Inflammatory Arthritis
Source: Front Immunol. 2021 Apr 27;12:669856. doi: 10.3389/fimmu.2021.669856 (PMC8110912; doi:10.3389/fimmu.2021.669856)
Supplement: Supplementary Figure 1 — Gating strategy to identify antigen experienced endogenous CD4 T cells from pLNs and joints. Representative flow cytometry plots identifying antigen experienced endogenous CD4 T cells (CD4+, CD45+, C45.1-, CD44hi) from (A) inflamed pLNs, and (B) inflamed joints at the early phase of mice undergoing inflammatory arthritis. [file DataSheet_1.pdf]

Sup fig 1

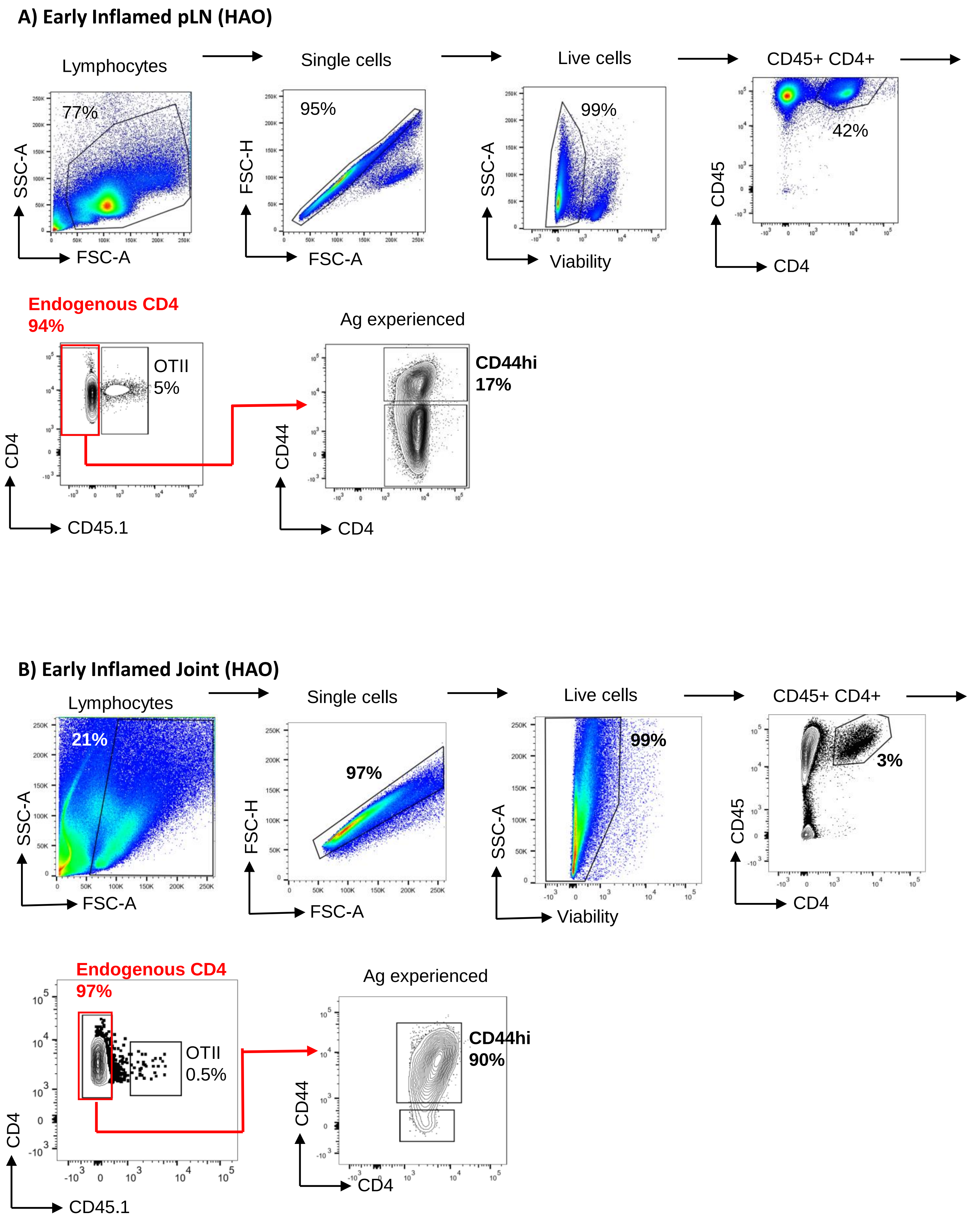

A) Early Inflamed pLN (HAO)
Lymphocytes
77%
SSC-A
FSC-A
Single cells
95%
FSC-H
FSC-A
Live cells
99%
SSC-A
Viability
CD45+ CD4+
42%
CD45
CD4
Endogenous CD4
94%
OTII
5%
CD4
CD45.1
Ag experienced
CD44hi
17%
CD44
CD4
B) Early Inflamed Joint (HAO)
Lymphocytes
21%
SSC-A
FSC-A
Single cells
97%
FSC-H
FSC-A
Live cells
99%
SSC-A
Viability
CD45+ CD4+
3%
CD45
CD4
Endogenous CD4
97%
OTII
0.5%
CD4
CD45.1
Ag experienced
CD44hi
90%
CD44
CD4

Sup fig 2

A) Early control pLN (PBS)

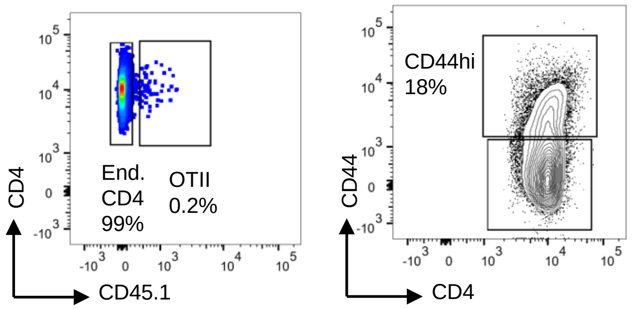

B) Early control joint (PBS)

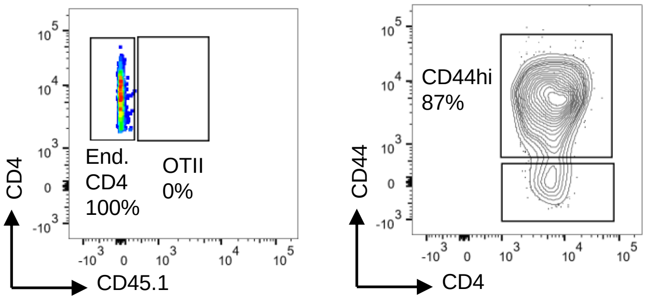

C) Late control pLN (IFA)

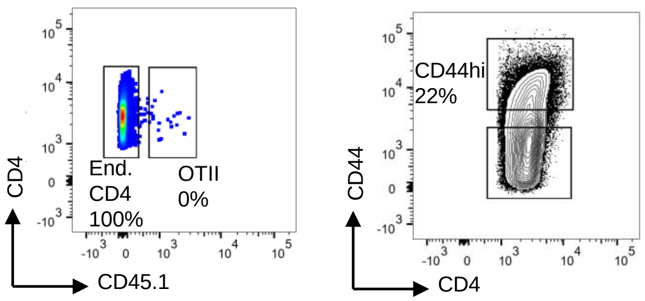

D) Late control joint (IFA)

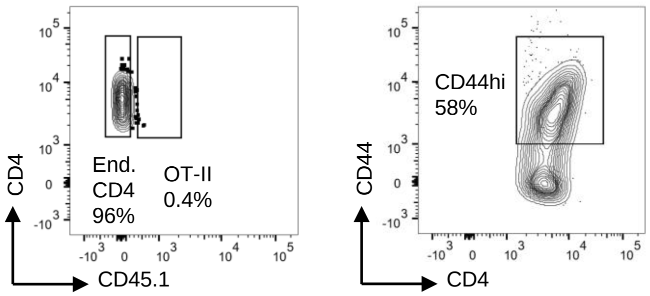

E) Late inflamed pLN (HAO+IFA)

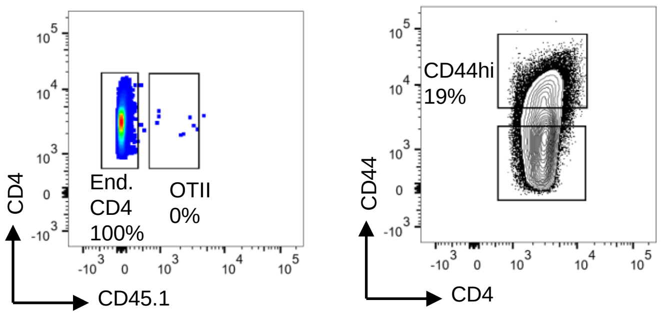

F) Late inflamed joint (HAO+IFA)

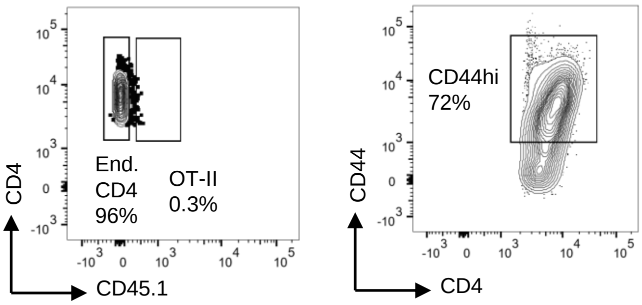

Sup fig 3

A)

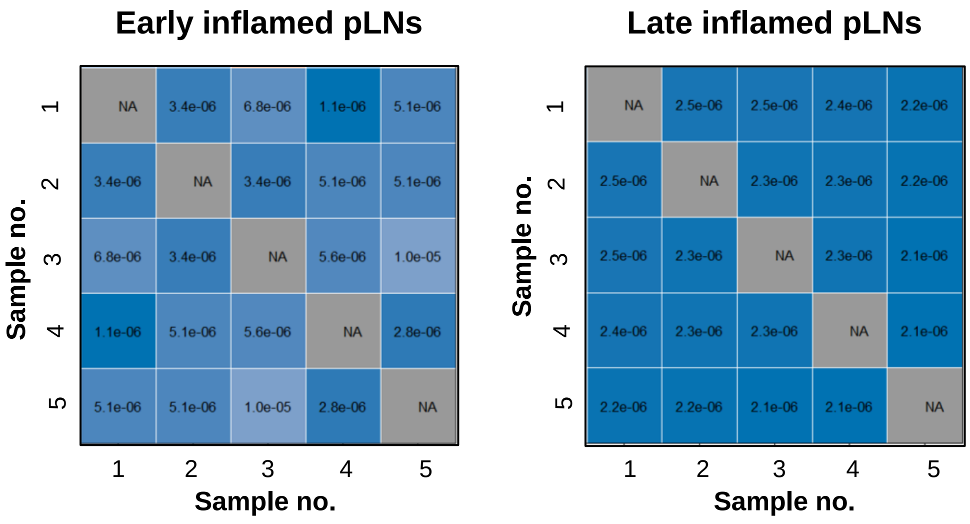

B)

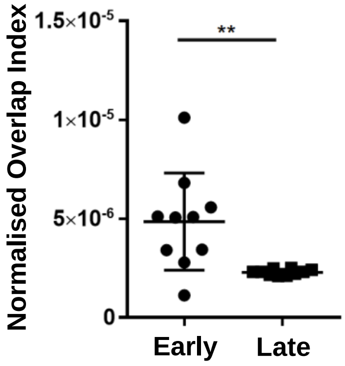

C)

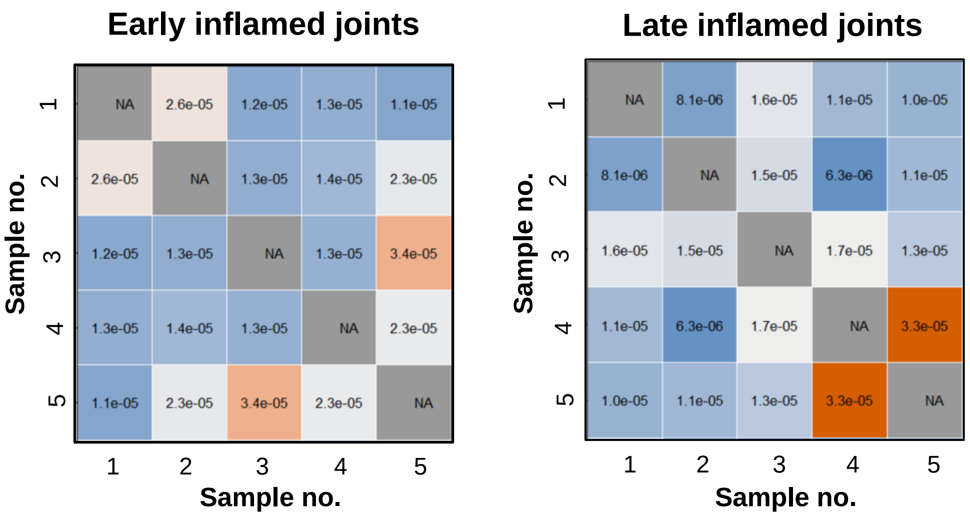

D)

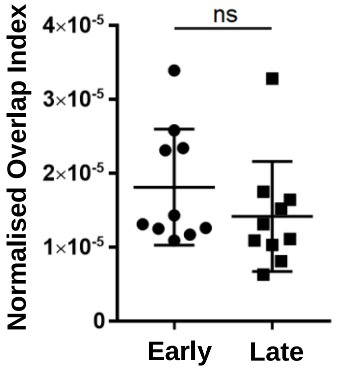

Sup table 1

Sample	CDR3β amino acid sequence	V-gene	Read count in pLN	Rank in pLN	Rank in joint
Top 10 early joint 1 in early pLN 1	CASSPGQGTGERLFF	TRBV26	4276	2	1
	CASSQTGGSYEQYF	TRBV2	275	95	7
	CASSNRDWGQDTQYF	TRBV16	267	98.5	3
	CASSFAGHYEQYF	TRBV3	94	196	5
Top 10 early joint 2 in early pLN 2	CASSLVGEDTQYF	TRBV16	1727	2	9
	CASSPQGNYAEQFF	TRBV2	1705	3	4
	CASSPGGGNYAEQFF	TRBV2	1384	6	10
	CASSLVQNTGQLYF	TRBV16	1004	15	7
	CASSRTGGNSDYTF	TRBV2	628	39	1
	CASSLGAYAEQFF	TRBV3	616	40	8
	CASSKLGGLSYEQYF	TRBV19	340	98	3
Top 10 early joint 3 in early pLN 3	CASSPLGGRNTGQLYF	TRBV5	2307	2	1
	CASSLGGESQNTLYF	TRBV12-2	1250	11	8
	CASSNREGTQYF	TRBV16	753	36	3
	CGAGQGNTEVFF	TRBV20	101	193	9
Top 10 early joint 4 in early pLN 4	CASSLGGESQNTLYF	TRBV12-2	14841	1	2
	CASSQGTGGQDTQYF	TRBV2	280	129	3
	CASSFGLEGQNTLYF	TRBV3	228	154	1
	CTCSARGSNTEVFF	TRBV1	209	174	6
	CASSPGQGVNTEVFF	TRBV4	61	506	8
Top 10 early joint 5 in early pLN 5	CASSQAGGYAEQFF	TRBV2	631	45	1
	CASSQTGVNAEQFF	TRBV2	250	153.5	5
	CASSQNTGQLYF	TRBV16	198	198.5	3

Sup table 2

Sample	CDR3β amino acid sequence	V-gene	Read count in pLN	Rank in pLN	Rank in joint
**Top 10 late joint 1 in late pLN 1**	CASSLDLGGRGEQYF	TRBV16	976	2	7
	CASSQTGGAREQYF	TRBV2	849	3	3
	CASSRQGNSDYTF	TRBV12-2	807	4	1
	CASSLSGLGGGAEQFF	TRBV26	764	5	2
	CASGGSGTGETLYF	TRBV13-2	456	7	4
	CASSQAQNTEVFF	TRBV2	330	11	8
	CASSQTGANTEVFF	TRBV2	153	38	10
	CASSLSLGGLEQYF	TRBV26	142	47	5
	CASSSLRGSNQAPLF	TRBV16	95	98	6
**Top 10 late joint 2 in late pLN 2**	CASSFSGQTEVFF	TRBV14	530	1	1
	CASSWGQGRDTQYF	TRBV16	267	6	3
	CASGDLDKYEQYF	TRBV13-2	167	18	2
	CTCSTHSDYTF	TRBV1	90	69.5	7
	CASSQNGALYEQYF	TRBV2	89	71.5	5
	CASSLQSSAETLYF	TRBV16	85	77.5	10
	CASQFRQDNYAEQFF	TRBV14	84	81	9
	CASSPPYINSDYTF	TRBV3	60	171	4
	CASSQAGGYAEQFF	TRBV2	52	226.5	6
	CASSKGPYNSPLYF	TRBV19	26	950	8
**Top 10 late joint 3 in late pLN 3**	CASSLETGGASEQYF	TRBV12-2	879	1	2
	CASRTASGNTLYF	TRBV19	798	2	4
	CARSTRAYNSPLYF	TRBV19	257	7	7
	CASSSNTGQLYF	TRBV16	217	12	1
	CTCSASGFSNERLFF	TRBV1	199	16	6
	CASSQTGGSYEQYF	TRBV2	164	25	5
	CASSQDSGGRAEQFF	TRBV2	158	31	8
	CASGRNYAEQFF	TRBV13-2	134	39	10
	CASSQDTGGLNTLYF	TRBV2	107	51.5	3
	CASSQPGTNQAPLF	TRBV2	52	197	9
**Top 10 late joint 4 in late pLN 4**	CASSKPGYAEQFF	TRBV19	435	3.5	1
	CASSQEAGRNTGQLYF	TRBV5	435	3.5	10
	CASSQTGAETLYF	TRBV5	260	14	4
	CASSRGPYNSPLYF	TRBV19	203	20	7
	CASSQAGGYAEQFF	TRBV2	170	26.5	2
	CASSSNTGQLYF	TRBV16	109	59.5	3
	CASSLAGGGYAEQFF	TRBV3	102	68	5
	CASSFGSSAETLYF	TRBV29	55	209.5	6
	CASSGQGWSTGQLYF	TRBV2	35	528	8
	CASSDRASSYEQYF	TRBV3	32	621.5	9
	CASSSNTGQLYF	*TRBV16*	*6*	*8460*	*3*
	CASSLAGGGYAEQFF	*TRBV3*	*1*	*30109.5*	*5*
**Top 10 late joint 5 in late pLN 5**	CASSLAWGGMAEQFF	TRBV16	515	3	4
	CASSHRLANSDYTF	TRBV14	496	4	1
	CTCSRDRGSGNTLYF	TRBV1	255	13	3
	CASSSNQDTQYF	TRBV16	217	19	7
	CASSPLGNYAEQFF	TRBV2	196	23	5
	CASSPPGQNQAPLF	TRBV2	76	95.5	6
	CASSQMTISNERLFF	TRBV2	76	95.5	10
	CASSLDGRSQNTLYF	TRBV16	34	377	8
	CASSQGQQDTQYF	TRBV2	34	377	9
	CASSQGQQDTQYF	*TRBV2*	*5*	*10330*	*9*
	CASSQGQQDTQYF	*TRBV2*	*3*	*17765.5*	*9*
	CASSQGQQDTQYF	*TRBV2*	*1*	*39595*	*9*
